# Repositioning of Anthelmintic Drugs for the Treatment of Cancers of the Digestive System

**DOI:** 10.3390/ijms21144957

**Published:** 2020-07-13

**Authors:** Federica Laudisi, Martin Marônek, Antonio Di Grazia, Giovanni Monteleone, Carmine Stolfi

**Affiliations:** 1 Department of Systems Medicine, University of “Tor Vergata”, 00133 Rome, Italy; federica.laudisi@gmail.com (F.L.); adigrazia2000@yahoo.it (A.D.G.); gi.monteleone@med.uniroma2.it (G.M.); 2 Institute of Molecular Biomedicine, Faculty of Medicine, Comenius University, 811 08 Bratislava, Slovakia; martin.maronek@gmail.com; 3 Division of Clinical Biochemistry and Clinical Molecular Biology, University of Rome “Tor Vergata”, 00133 Rome, Italy

**Keywords:** drug repurposing, colorectal cancer, hepatocellular carcinoma, chemotherapy, benzimidazole, salicylanilide, niclosamide, rafoxanide, STAT3, Wnt/β-catenin

## Abstract

Tumors of the digestive system, when combined together, account for more new cases and deaths per year than tumors arising in any other system of the body and their incidence continues to increase. Despite major efforts aimed at discovering and validating novel and effective drugs against these malignancies, the process of developing such drugs remains lengthy and costly, with high attrition rates. Drug repositioning (also known as drug repurposing), that is, the process of finding new uses for approved drugs, has been gaining popularity in oncological drug development as it provides the opportunity to expedite promising anti-cancer agents into clinical trials. Among the drugs considered for repurposing in oncology, compounds belonging to some classes of anthelmintics—a group of agents acting against infections caused by parasitic worms (helminths) that colonize the mammalian intestine—have shown pronounced anti-tumor activities and attracted particular attention due to their ability to target key oncogenic signal transduction pathways. In this review, we summarize and discuss the available experimental and clinical evidence about the use of anthelmintic drugs for the treatment of cancers of the digestive system.

## 1. Introduction

Cancer represents a major public health and economic problem and is a leading cause of death worldwide. For both sexes combined, lung and breast cancer are the most commonly diagnosed cancers (accounting for 11.6 % of the total cases each) and the leading causes of cancer death (18.4% and 6.6% of the total cancer deaths respectively) [1]. Nevertheless, cancers of the digestive system, when combined together, account for more new cases and deaths per year than cancers arising in any other organ [1]. In 2018, among all the cancers of the digestive system, colorectal cancer (CRC) was estimated to have both the highest number of new diagnosed cases and the highest number of deaths followed by stomach and liver cancer [1]. Present day therapy of solid tumors is focused on surgical removal, although this option is usually available only in the earlier stages of cancer development [2]. In later stages, when metastases are detected, chemotherapy is the treatment of choice. Unfortunately, many patients experience chemotherapy resistance, either from the beginning or during the course of treatment, ultimately resulting in therapy failure [3]. These issues create the need for other therapeutic options and force the pursuit of the discovery of new chemicals.

However, the development of new therapeutics has become increasingly difficult for pharmaceutical companies, as the pipeline leading to any drug’s development is extremely protracted, exacting and costly. Nowadays, it takes about 10–15 years to develop a new drug [4], and despite all the effort, the success rate remains very low (around 2 %) [5]. In recent years, the pool of newly discovered and Food and Drug Administration (FDA)-approved drugs has been dwindling [6], mostly due to the increasing cost of clinical trials [7]. From 2004 through 2012, the median total cost for a pharmaceutical clinical trial was estimated to be around 2–3 billion US dollars, although, when adjusted for inflation, the money required for funding would be even higher by now [4]. It has been predicted that for each dollar invested, less than a dollar returns as profit, thereby making the development of new drugs a risky and undesirable process for most companies [8]. Given these drawbacks, new approaches to drug development should be employed in order to make drug research less time-consuming and financially demanding.

One such approach is drug repositioning (also referred to as drug repurposing); that is, the strategy of identifying new uses for approved or investigational drugs that are outside the scope of the original medical indication [9].

Drug repositioning presents a number of advantages over the development of new therapeutic agents. As mentioned, one advantage lies in the lower cost as compared to the traditional developing of a new drug. Indeed, while all of the steps and trials required to design, test and implement a new drug in the pharmaceutical industry may reach as much as 13 billion dollars [10], drug repositioning offers a much more affordable cost (bringing a repurposed drug to market has been estimated to cost 300 million US dollars on average) [11]. It needs to be mentioned, however, that the cost-saving estimates vary quite considerably. For example, another source estimates the cost reduction to be around 50–60% [12]. Even more importantly, the time needed for translating the approval into a possible therapy is much shorter, as old drugs with the potential for repositioning have already passed both clinical trials and FDA-approval. This results not only in a speed-up of the entire process but also in higher predicted safety as compared to a new drug [9,12].

**Figure 1:**
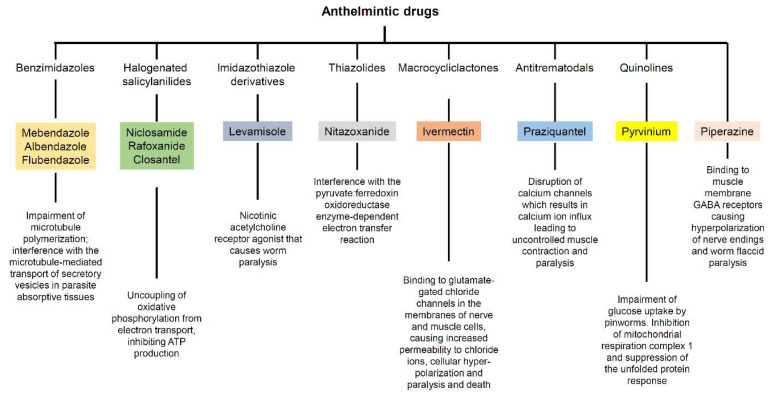
Class and mechanism of action against parasites of the anthelmintic drugs reviewed herein.

Anthelmintic drugs are a group of agents that act against infections caused by parasitic worms (helminths) that colonize the human intestine. These drugs were initially developed for treating veterinary parasites and have subsequently advanced from treatment of livestock to clinical applications for patients. Anthelmintic drugs encompass various chemical entities and have several distinct modes of action (Figure 1).

Among the drugs investigated for repurposing in oncology, a relevant number are anthelmintics, some of which have been used in the clinic for decades. The serious consideration of such drugs as possible anti-cancer agents somewhat relies on their ability to interfere with crucial oncogenic pathways (e.g., Wnt/β-catenin, STAT3 and NF-κB). In this context, it is worth mentioning the emerging role of mitochondria in mediating the anti-tumor effects of anthelmintic compounds, although most of the witnesses have been produced in blood, renal and gynecologic tumors [13,14,15,16,17,18,19,20].

Here, we review and discuss the available experimental and clinical evidence about the use of anthelmintic drugs for the treatment of cancers of the digestive system.

## 2. Anti-Cancer Effects of Anthelmintic Drugs in Malignancies of the Digestive System

In the last two decades, anthelmintic drugs have gained attention as possible anti-cancer agents in malignancies of the digestive apparatus [21,22]. In particular, the cytostatic/cytotoxic effect of many anthelmintic drugs, such as those belonging to benzimidazoles and salicylanilides, has been already demonstrated in vitro and in preclinical models. Additionally, a number of anthelmintic drugs have been employed in clinical trials, some of which are still ongoing.

Such drugs are summarized in Table 1 and Table 2 respectively and discussed below.

### 2.1. Benzimidazoles

Benzimidazoles are a group of heterocyclic aromatic organic compounds that include important anthelmintic drugs (e.g., mebendazole, albendazole, thiabendazole, fenbendazole, triclabendazole, flubendazole). In the last few years, some of them have been successfully tested for the treatment of gastrointestinal, liver and pancreatic cancers.

#### 2.1.1. Mebendazole

Mebendazole (MBZ) is an anthelmintic drug commonly used to treat groups of parasitic worms (e.g, roundworms, hookworms and pinworms). In particular, by blocking tubulin polymerization, MBZ is able to compromise microtubule function in the intestinal cells of the helminths, and consequently, their nutrient uptake. The anti-cancer properties of MBZ for the treatment of gastrointestinal cancers have been intensively studied over the last decade. For example, MBZ was seen to inhibit cell growth and invasion of a human malignant ascites cell line derived from a primary gastric tumor, whether used alone (0.15–20 μM) or in combination with the chemotherapeutic drug 5-fluorouracil (5-FU) [23]. Moreover, MBZ treatment was also successfully tested in five CRC cell lines (IC50 < 5 μM), whereas it showed no cytotoxic activity against three cell lines with non-malignant phenotype [24]. In vivo, MBZ anti-cancer efficacy was tested in a model of familial adenomatous polyposis (FAP), using mice carrying a constitutional mutation in the *Adenomatous polyposis coli* (*Apc*) gene (*Apc*^min/+^ mice [25]. In particular, MBZ (35 mg/kg) decreased the number and size of intestinal microadenomas once used in combination with the non-steroidal anti-inflammatory drug sulindac [25]. Mechanistically, these two drugs were able to inhibit both MYC and COX2 pathways, and angiogenesis and the release of pro-tumorigenic cytokines [25]. Notably, in a case report by Nyger and Larsson, MBZ induced remission of lung and lymph node metastases, and a partial remission of liver metastases, in a patient with refractory metastatic colon cancer [26].

MBZ showed anti-cancer properties in the treatment of hepatocellular carcinoma (HCC) as well. In particular, MBZ (0.5–5 μmol/L) negatively targeted the MAPK pathway—by reducing the expression of p-MEK1/2 and p-ERK1/2—whether used alone or in combination with the chemotherapeutic drug sorafenib, and improved liver function (when administered at 100 mg/kg/day) in an animal model of HCC [27]. MBZ is currently being employed in clinical trials for the treatment of gastrointestinal cancers. A phase 2a study about the safety and efficacy of MBZ in patients with advanced gastrointestinal cancer was recently terminated because of lack of efficacy (NCT03628079), whereas another study testing MBZ as adjuvant therapy in CRC patients is now recruiting patients (NCT03925662) (Table 2).

#### 2.1.2. Albendazole

Albendazole (ABZ) is another important benzimidazole compound used as anthelmintic drug in the treatment of several parasitic infections, such as giardiasis, ascariasis, trichuriasis and filariasis. The mechanism of action of ABZ shows similar features to MBZ, as it is able to affect the microtubule polymerization in the intestinal cells of the worms.

Preclinical investigations have suggested that ABZ might be useful for the treatment of some cancers of the digestive system. For instance, Mohammad et al. observed a significant cytostatic effect of ABZ (0.01—10 μM) on the human CRC cell line HT-29 in vitro, and a marked reduction of tumor growth in nude mice bearing peritoneal HT-29-derived xenografts following intraperitoneal ABZ administration (150 mg/kg) [28]. Further evidence confirmed the anti-proliferative effect of ABZ (0.25 and 0.5 μmol/L) in additional intestinal cancer cell lines (i.e., SW480, SW620, HCT8 and Caco2), especially when used in combination with the chemotherapeutic agent Paclitaxel [29]. ABZ has attracted attention as a potential anti-cancer agent also for the treatment of HCC [30]. In particular, ABZ was reported to hamper the growth of rat, mice and human HCC cells in a dose-dependent manner [30]. Such an effect associated with a block of the cell cycle at the G0/G1 phase at lower ABZ concentrations (250 nM), whereas cells treated with higher ABZ concentrations (1000 nM) showed a transition delay through the G2/M phase [30]. In vivo, oral ABZ treatment (300 mg/kg) of nude mice inoculated subcutaneously with SK-HEP-1, a human hepatic adenocarcinoma cell line, markedly suppressed tumor growth [30].

Comparable results have been recently obtained in pancreatic cancer. ABZ was seen to negatively affect proliferation, migration and viability of the human pancreatic cancer cell lines SW1990 and PANC-1 in a dose-dependent manner [31]. Moreover, ABZ exerted its cytostatic effect also in vivo in a nude mouse xenograft model, wherein it significantly reduced tumor growth [31].

Some of the above-mentioned experimental evidence encouraged the scientific community to clinically test ABZ in CRC and HCC patients. A pilot study was conducted in a small number of patients (*n* = 7) over a period of 28 days [63]. ABZ (10 mg/kg) was well tolerated except for some cases of neutropenia (*n* = 3), and most of the patients (*n* = 5) showed complete or partial reduction of some tumor markers [63]. However, further studies with an adequate number of recruited patients are required to validate such observations.

#### 2.1.3. Flubendazole

Another benzimidazole compound with potential anti-cancer activity is flubendazole (FLU). In addition to its ability to combat gastrointestinal nematode infections, FLU (0.1—10 μMol/L) was reported to inhibit CRC cell growth and to synergize, similarly to ABZ, with Paclitaxel [29]. Further studies revealed that FLU (1 µM) exerted its anti-mitogenic effect on the CRC cell lines SW480 and SW620 by altering cyclin B1 and cyclin D1 levels. In the same cells, FLU induced apoptosis and premature senescence [32], and impaired cell adhesion by reducing the expressions of several adhesion markers (ICAM-1; αE-catenin; β-catenin; integrin α5 and β1) [33]. In addition, FLU negatively affected the phosphorylation/activity of nuclear factor-kappa B (NF-κB), a transcription factor whose activation modulates cell proliferation, survival and metastatic potential in transformed cells [64]. FLU also suppressed the expression of metastatic markers (ICAM-1, EpCAM, integrin α5, β1, α-tubulin), thereby blocking cell migration [33]. However, despite that promising experimental evidence, no clinical studies employing FLU have been performed so far.

### 2.2. Halogenated Salicylanilides

The halogenated salicylanilides are a group of anthelmintic drugs commonly used to treat intestinal tapeworm and fluke infections. These compounds consist of a salicylic acid ring and an anilide ring and include a wide range of drugs, such as niclosamide, rafoxanide and closantel.

#### 2.2.1. Niclosamide

Niclosamide is a Food and Drug Administration (FDA)-approved drug for the treatment of tapeworm infections and whose mechanism of action consists of uncoupling the oxidative phosphorylation. In the last decade, niclosamide has gained the interest of the scientific community due to a large body of evidence indicating this drug as a potent inhibitor of key oncogenic pathways [22].

In particular, niclosamide (0.4, 2 and 10 μMol/L) was reported to inhibit the Wnt/β-catenin signaling pathway, whose aberrant activation occurs in approximately 80% of sporadic CRCs [65], and proliferation of the human CRC cell lines HCT-116, HT-29 and Caco2, regardless of mutations in the key regulator APC [34]. The diminished β-catenin signaling was linked to the ability of niclosamide to down-regulate the expression of the Wnt pathway component dishevelled-2 (Dvl2). Moreover, niclosamide-mediated anti-cancer effects were seen in primary CRC cells isolated by surgical resection from the livers of patients with metastatic disease and in NOD/SCID mice (when used at 200 mg/kg) implanted with human CRC xenografts [34]. Recently, elegant studies by Wang and colleagues showed that the inhibition of the Wnt/β-catenin cascade by Niclosamide (used at 0.04–10 μM) was mediated by the induction of autophagy [35].

Along the same lines was the work of Monin and colleagues in human (SW480, SW620) and rodent (CC531) CRC cells. The authors reported the ability of Niclosamide (1, 3 and 10 μM) to alter both canonical and non-canonical Wnt-signaling pathways by affecting the formation of the β-catenin-Bcl9-LEF/TCF triple-complex and inducing the expression of the proto-oncogene c-jun [36].

At the time of this review, three clinical trials aimed at investigating the anti-cancer effects of niclosamide in CRC patients are ongoing (Table 2). The first started in 2017 to test niclosamide’s efficacy in patients with resectable colon cancer, but no results are available so far due to low enrolment rate (NCT02687009). The other two clinical studies, instead, have just started the recruiting phase and aim at testing the anti-cancer effects of niclosamide in patients with metastases of CRC progressing after therapy (NCT02519582) and with FAP (NCT04296851) respectively.

Anti-cancer activities of niclosamide were also seen in HCC. Weng et al. reported that niclosamide suppressed HCC cell growth and triggered apoptotic cell death by inducing endoplasmic reticulum (ER) stress [37]. In depth, such effects were associated with the induction of reactive oxygen species (ROS) and relied on the activation of PKR-like ER kinase (PERK) and ATF3 [37]. In another study, niclosamide (1–20 μM) was shown to impair proliferation and to induce apoptosis in multiple HCC cell lines (i.e., HepG2, QGY-7703 and SMMC-7721) by negatively affecting the phosphorylation/activity of the oncogenic transcription factor STAT3 [38]. In the same cells, niclosamide was also able to potentiate the cytotoxicity of the chemotherapeutic drug cisplatin [38].

Alasadi and colleagues tested the anti-cancer efficacy of niclosamide ethanolamine (NEN), the ethanolamine salt of niclosamide, which has a similar safety profile to niclosamide but is better adsorbed in the liver after oral administration [40]. NEN (0.5–5 μM) was protective against HCC whether used alone or in combination with oxyclozanide, another anthelmintic drug with mitochondrial uncoupling activity [40]. In particular, both drugs induced HCC cell arrest in the G0/G1 phase and impaired cell migration and invasion [40]. Additionally, chow-administered NEN (1500 ppm, resulting in 120–150 mg/kg/day) significantly reduced intestinal polyp formation in *Apc*^min/+^ mice and decreased, together with oxyclozanide, the number of hepatic metastases in a mouse model of CRC metastasis (NEN given at 2000 ppm with chow) [40]. Altogether, the above-mentioned experimental evidence may pave the way to future clinical trials aimed at investigating the safety and efficacy of niclosamide and its derivates also in patients affected by HCC.

More recent studies showed a protective effect of niclosamide in esophageal cancer [39]. In particular, niclosamide (2.5–10 μM) suppressed the STAT3 signaling pathway in esophageal adenocarcinoma cells (BE3) and esophageal squamous cell carcinoma cells (CE48T and CE81T) by impairing both STAT3 phosphorylation on Y705 residue and STAT3 protein expression. This effect resulted in the arrest of cells in the G0/G1 phase and induction of cell apoptosis [39]. Moreover, niclosamide potentiated the cytotoxic effects of some chemotherapeutic drugs, such as 5-FU, cisplatin and paclitaxel, thereby shifting their IC50 to lower doses with the ultimate result of reducing potential side effects in treated patients [39].

#### 2.2.2. Rafoxanide

Rafoxanide, another halogenated salicylanilide, is approved by the FDA for the veterinary treatment of fascioliasis and some gastrointestinal roundworms [66,67,68]. Although evidence regarding the usage of Rafoxanide in humans is poor, a study reported the therapeutic use of the drug in a seven-year-old girl affected by fascioliasis [69]. Rafoxanide showed a markedly lower hemolytic activity compared to niclosamide [70] and was reported to be a potent inhibitor of the oncogenic BRAF V600E mutant protein, commonly found in melanomas and CRCs and associated with a poorer prognosis for patients [71]. Recent studies in CRC from our group showed that rafoxanide (1.25–5 μM) restrained the growth of human CRC cells—bearing both the mutated V600E (HT-29) and wild-type BRAF alleles (HCT-116 and DLD-1)—but not that of normal colonic epithelial cells [41]. Rafoxanide’s anti-mitogenic ability relied on selective induction of the ER stress in CRC cells and was associated with cyclin D1 protein down-regulation, accumulation of cells in the G0/G1 phase and subsequent caspase-dependent apoptosis [41]. These observations were also confirmed in ex vivo human CRC explants [41]. In addition, we tested the ability of rafoxanide to inhibit intestinal tumor development in vivo using *Apc*^min/+^ mice treated with the carcinogen azoxymethane, a model that mimics human sporadic CRC. Rafoxanide (7.5 mg/kg) arrested colon carcinogenesis once systemically administered to *Apc*^min/+^ mice, leading to reduced number and size of neoplastic lesions in treated animals. Notably, rafoxanide seemed well-tolerated as no significant changes in body weight were observed in mice treated with the drug as compared with sham [41]. More recently, our follow-up studies indicated Rafoxanide as a bona fide immunogenic cell death (ICD) inducer in CRC cells [42]. In detail, in HCT-116 and DLD1 CRC cells, rafoxanide (1.25–5 μM) induced autophagy and all the main damage-associated molecular patters (DAMPs) (ecto-calreticulin exposure, adenosine triphosphate (ATP)/high mobility group box 1 (HMGB1) release) required for ICD. In vivo, rafoxanide markedly reduced tumor growth, as compared with sham, in a vaccination assay where immunocompetent mice were immunized with rafoxanide-treated syngeneic dying tumor cells before being re-challenged with living cells [42]. Rafoxanide’s anti-cancer effects, similarly to those reported above, were described in gastric cancer [43]. Indeed, Rafoxanide arrested the proliferation of gastric cancer cells in the G0/G1 phase of the cell cycle, and induced autophagy and apoptosis through the inhibition of the PI3K/Akt pathway both in vitro (12.5–100 μM) and in vivo (20 mg/kg) [43].

Collectively, these data suggest that Rafoxanide could potentially be deployed, whether alone or in combination with other therapeutics, as anti-cancer agent in patients affected by CRC and gastric cancer. However, further studies of dosage and long-term toxicity are needed to confirm the therapeutic potential and clinical benefit of the drug.

#### 2.2.3. Closantel

Closantel is another halogenated salicylamide agent reported to inhibit BRAF V600E [71]. Closantel (0.025–10 µM) showed anti-angiogenic and anti-tumor properties in zebrafish and significantly inhibited cell proliferation in zebrafish xenotransplanted with human tumor cells, including liver and pancreatic cancer cells [44]. However, further studies in experimental and preclinical models are required to confirm closantel’s anti-cancer effects and provide new clues about its possible clinical application.

### 2.3. Other Anthelmintic Drugs

Besides drugs belonging to benzimidazole and halogenated salicylanilide groups, other anthelmintic drugs have been proposed as potential anti-cancer agents. Such drugs are reported and discussed below.

#### 2.3.1. Levamisole

Levamisole belongs to the family of imidazothiazoles, which target the roundworm’s nervous system and paralyze the parasite’s muscles by acting as nicotinic acetylcholine receptor agonist [72]. Levamisole attracted interest for its immunomodulatory properties due its ability to promote innate and adaptive immune responses, especially in depressed immune subjects [73]. The drug was also proposed as an anti-cancer agent for CRC treatment in the early 1990s, when it was mainly used as an adjunctive agent to standard cytotoxic agents, such as 5-FU [60]. However, levamisole monotherapy has never been considered due to its inability to directly affect cancer cell proliferation and/or survival. Levamisole was tested in several clinical trials (Table 2). The first large-scale study was the Intergroup trial (INT-0035) in 1990, wherein patients with stage III CRC underwent surgery with or without treatment for 12 months with levamisole or surgery plus treatment for 12 months with 5-FU plus levamisole [60]. Results showed a significant reduction of recurrence and death rates in patients receiving combined therapy [60]. However, these promising data were not confirmed in another large randomized study, wherein 6-month treatment with 5-FU plus levamisole did not improve patient survival as compared to a combination treatment with 5-FU plus leucovorin [61]. Along the same lines was a later study by Wolmark et al. [62], wherein 1-year of levamisole treatment gave no additional benefits to the regimen with 5-FU plus leucovorin in CRC patients.

Taken together, the above results do not support the usage of levamisole as adjunct treatment for CRC. However, they do not rule out the possibility of employing this drug for the management of other tumors of the digestive apparatus. Currently, two phase III clinical trials are testing the efficacy and safety of levamisole (Table 2). The first one aims at evaluating levamisole in combination with arginine hydrochloride for the treatment of patients with advanced HCC (NCT03950518). The second one was set up to test levamisole hydrochloride for the treatment of patients with advanced intrahepatic cholangiocarcinoma (NCT03940378).

#### 2.3.2. Nitazoxanide

Nitazoxanide (NTZ) belongs to the thiazolide family and is effective against intestinal parasites (e.g., *Cryptosporidium parvum* and *Giardia lamblia*); *Helicobacter* and *Clostridium*-driven bacterial infections; and viral infections [74,75,76,77,78]. NTZ is considered a promising candidate for cancer treatment due to a large body of evidence reporting anti-proliferative and pro-apoptotic activities against different cancer types [45,46,47,48] and its excellent pharmacokinetic and safety profiles. Concerning CRC, NTZ (50 µM) was shown to induce apoptosis in CRC cells in a glutathione-*S*-transferase P1 (GSTP1)-dependent manner [45]. In HCT-116 and HT-29-derived spheroids, NTZ activated the AMPK pathway and down-regulated c-Myc, mTOR and Wnt signaling at clinically achievable concentrations (0.1–17 μmol/L) [46]. These observations were confirmed in vivo, wherein the combined therapy of NTZ (100 mg/kg) with the chemotherapeutic drug irinotecan (40 mg/kg) strongly suppressed tumor growth in a mouse xenograft model as compared to irinotecan alone [46].

A following study further confirmed the anti-cancer activity of NTZ against CRC, indicating the drug as a potent inhibitor of Wnt/β-catenin signaling pathway [47]. Mechanistically, NTZ (10 µM) directly targeted the peptidyl arginine deiminase 2, leading to increased citrullination and degradation of β-catenin in CRC cells [47]. More recently, Ripani and co-workers tested the anti-cancer efficacy of the nitazoxanide bromo-derivate RM4819 [48]. Although RM4819 (10 µM) did not show the anti-bacterial activity of NTZ, it could arrest the cell cycle and uncouple the mitochondrial respiration as NTZ did. Moreover, RM4819 strongly suppressed the activity of mitochondrial complex III, thereby inducing relevant energy deficiency in CRC cells [48]. Similar results were obtained in intestinal organoids, wherein both NTZ and RM4819 inhibited the proliferation of intestinal tumoroids, without affecting normal intestinal organoids [48]. Despite the encouraging evidence, no clinical trials are currently ongoing to test the safety and efficacy of NTZ and its derivates in CRC patients.

#### 2.3.3. Ivermectin

Ivermectin is a macrocyclic lactone belonging to the group of avermectins [79]. Ivermectin is commonly used in veterinary medicine and in the clinic for the treatment of onchocerciasis and lymphatic filariasis, due to its ability to increase the activity of γ-aminobutyric acid (GABA) receptors or glutamate-gated chloride ion channels (Glu-Cl) to block the parasite’s nerve–muscle signaling [80].

Ivermectin was reported as a potent inhibitor of the Wnt-TCF pathway in CRC cells [49]. In particular, at low micromolar concentrations (1–5 μM), ivermectin suppressed the expression of Wnt-TCF targets, such as AXIN2, LGR5 and ASCL2 in DLD1 and Ls174T CRC cell lines and blocked colon cancer stem cell self-renewal in a clonogenic assay [49]. Moreover, it repressed β-catenin phosphorylation and cyclin D1 expression in an okadaic acid-sensitive manner, indicating the involvement of protein phosphatases in such actions [49]. In vivo, ivermectin (10 mg/kg) impaired the growth of DLD1 and HT-29-derived xenografts in nude mice in a TCF-dependent fashion without obvious side effects [49].

Nambara and colleagues reported important anti-tumor effects for ivermectin in the treatment of gastric cancer [50]. In particular, ivermectin suppressed MKN1 gastric cancer cell growth in vitro and in vivo (when used at 10 µM and 10 mg/kg respectively) through the inhibition of the nuclear expression of yes-associated protein 1 (YAP1) [50], which plays a key role in gastric carcinogenesis [81].

Finally, ivermectin-related anti-cancer properties were reported in cholangiocarcinoma (CCA) [51]. Ivermectin treatment (0.5–32 µM) induced S-phase cell cycle arrest and apoptotic cell death in both gemcitabine-sensitive (KKU214) and gemcitabine-resistant (KKU214GemR) CCA cell lines [51], thereby suggesting this drug for the treatment of CCA patients who are resistant to gemcitabine.

#### 2.3.4. Praziquantel

Praziquantel, an anthelmintic drug particularly active against parasitic flatworms, has long been considered one of the best therapeutic options for the treatment of schistosomiasis [82]. Praziquantel’s anti-parasitic mechanism of action has not been established yet, but it could rely on the ability to block Ca^2+^ channels, with the ultimate result being to increase intracellular Ca^2+^ and induce muscular contractions [82]. Schistosomiasis development is known to be linked to the habit of eating raw fish, especially in countries from South-East Asia (e.g., Thailand and Vietnam). Recent studies have reported that patients who experienced repeated administrations of the drug are more likely to develop CCA as compared to untreated subjects [83]. However, praziquantel should not be considered as a direct causative agent for cholangiocarcinogenesis, as the recurrent parasite infection is considered to be the major risk factor for CCA development [83].

In 2012, Wu and colleagues provided evidence that praziquantel (20–40 µM) could potentiate the cytotoxic effects of paclitaxel [52]. In particular, the combined therapy could synergistically impair cell proliferation and induce apoptotic cell death in several cancer cell lines, including the CRC cell line DLD-1, which is known to be resistant to paclitaxel treatment [52]. Mechanistically, co-treatment with praziquantel and paclitaxel impaired the expression of XIAP, an anti-apoptotic protein which is a potent inhibitor of caspase activity [52].

#### 2.3.5. Pyrvinium

Pyrvinium is commonly used to treat pinworm infections in both animals and humans. Several pyrvinium derivatives have been generated, and among them, the salt pyrvinium pamoate (PP) was seen to be particularly efficient against cancer cells [84].

Early studies dating back to 2004 found that PP (0.1 and 1 μg/mL) was extremely toxic to the human pancreatic tumor cell line PANC-1 cultured under glucose deprivation but not in ordinary medium [53]. This effect was associated with the inhibition of Akt phosphorylation, a pro-survival mechanism triggered in response to various stresses, such as glucose starvation [53]. The anti-tumor activity of PP was confirmed in vivo in a hypovascular pancreatic cancer model, wherein immunocompromised mice were xenografted with PANC1 cells. Oral administrations of PP (100 and 200 μg) reduced tumor growth and inhibited Akt phosphorylation, although some tumor cells survived, probably because PP exerted its toxic effect mainly during glucose starvation [53]. In a subsequent paper, Yu et al. shed light on the possible mechanisms underlying the above-mentioned effects [54]. The authors provided evidence that PP (0.1 and 0.3 μM) restrained the transcriptional activation of GRP78 and GRP94, two key molecular chaperones involved in the unfolded protein response (UPR) induced by glucose deprivation [54]. Other UPR pathways induced by glucose starvation, such as XBP-1 and ATF4, were also suppressed by PP. Notably, ectopic GRP78 expression partially protected cells from PP-induced cell death under glucose starvation [54]. Xenograft experiments in nude mice transplanted with either HCT-116 or the pancreatic cancer cell line AsPC-1, showed rather marginal overall anti-tumor activity for PP (administered 10 mg/kg via oral gavage and 0.5 mg/kg intraperitoneally) as a monotherapy. However, the PP efficacy in reducing tumor growth was markedly enhanced by combination therapy with doxorubicin [54].

Later reports indicated a role for PP in the modulation of the Wnt/β-catenin pathway. In the CRC cell lines HCT-116 and SW480, which express mutated *Apc* or *β-catenin* genes, PP (10–100 nM) was found to inhibit the Wnt signaling by interacting with and activating casein kinase 1 alpha (CK1α), which in turn, bound to and phosphorylated β-catenin [55]. Moreover, PP could also affect the Wnt pathway by targeting and promoting the degradation of the Pygopus protein, a transcriptional component of Wnt [55]. Follow-up experiments in *Apc*^min/+^ mice showed that oral administration of PP (25 mg/kg) attenuated the levels of Wnt-driven biomarkers and induced apoptosis in transformed cells, thereby inhibiting adenoma formation [56]. A study by Wiegering and co-workers confirmed such experimental evidence and described a synergistic effect of PP (10^−7^ mol/L) on HCT-116 and SW620 cell lines, once combined with 5-FU [57]. Interestingly, PP failed to induce apoptosis in Colo741 cells, bearing no mutation in the Wnt pathway [57]. Finally, PP inhibited the migration of HCT-116 cells in vitro and delayed liver metastases formation in a xenograft model, wherein nude mice were injected with HCT-116 cells in the portal vein and then treated with PP (1 mg/kg) [57]. Overall, these results suggest that PP might be repurposed for the clinical treatment of pancreatic cancer and CRC, although no clinical trials are available at the moment.

#### 2.3.6. Piperazine

Piperazine was first used as an anthelmintic in the 1950s and it is still the active constituent of over-the-counter remedies for the treatment of worm infections in children. Piperazine binds directly and selectively to muscle membrane GABA receptors, presumably causing hyperpolarization of nerve endings, resulting in the paralysis of the worm [85]. The first study reporting anti-cancer effects of piperazine is dated back to 1963, when McNair and colleagues described an effect of piperazine derivatives: preventing tumor growth in rats grafted with Walker 256 carcinosarcoma cells [86]. Recent evidence has confirmed the ability of piperazine and its derivatives to exert important anti-neoplastic activity on cancer cells. For instance, the piperazine derivative AK301 (used at 5 µM) was tested on CRC cell lines (i.e., HT-29 and HCT-116); it dramatically impaired tubulin polymerization and induced mitotic arrest [58]. In parallel, AK301 increased the expression of tumor necrosis factor (TNF) receptor 1, thereby making cancer cells more susceptible to apoptotic cell death by TNF-α [58]. Another piperazine derivate, called BK10007S, was reported to exert anti-cancer effects on HCC cell lines [59]. BK10007S (0, 3, 7 and 8.5 μM) blocked HepG2 and SK-Hep1 cell proliferation upon reduction of cyclin D1 expression. Moreover, BK10007S (7 and 8.5 μM) induced apoptotic cell death by promoting caspase-3 and PARP-1 protein cleavage, and by decreasing the phosphorylation of AKT and ERK kinases and the expression of survivin and CUGBP1 proteins [59]. Collectively, such results suggest that piperazine derivates could potentially be deployed as anti-cancer agents in patients affected by CRC and HCC.

## 3. Discussion

The repositioning of approved and abandoned drugs is becoming a popular strategy in oncology drug development, representing a solution to the high costs of discovering new drugs, and due to the already approved safety and pharmacological profiles, a unique opportunity to expedite promising anti-cancer therapies to the clinic [87]. Besides these advantages, as most non-cancer drugs have minor or tolerable side effects, repurposing of such compounds in oncology may also elude the detrimental impacts of conventional cancer chemotherapeutics (mainly DNA damaging agents) on patients’ quality of life [88].

In the context of cancers of the digestive apparatus, drugs belonging to some classes of anthelmintics (exemplified in this review and summarized in Table 1) have shown, whether alone or in combination with other anti-cancer agents, pronounced anti-tumor activities in vitro and in preclinical models, and have attracted considerable interest due to their ability to target key oncogenic signal transduction pathways. Some of these drugs (summarized in Table 2) are, or have been, also under phase II/III clinical trials for cancer therapy. Despite all the enthusiasm, the efficacy in clinical trials—as commonly occurs with drugs repurposed in other contexts—remains the major bottleneck for the successful repositioning of anthelmintic agents in oncology [89]. This, in our view, may be in part due to limitations related to the in vitro experiments (often performed in cancer cell lines) commonly used to screen anthelmintics with potential anti-tumor activities, and to the heterogeneity of primary tumors and metastases [90,91]. Computer-based approaches—to predict novel drug-disease pairs—and the use of more robust phenotypic assays (e.g., patient-derived organoids, preclinical in vivo models) for the initial test of anthelmintics bearing potential anti-tumor actions, would likely contribute to selecting agents with higher potential for success in the clinic. Moreover, as most studies lack data on normal cells/tissues, future experimental work should take into account investigating the potential toxicity of the repurposed drugs on non-cancer cell populations (e.g., corresponding non-transformed cells, immune cells and stromal cells) following both short and long-term administration.

Other possible drawbacks for the successful repositioning of anthelmintic drugs in oncology relate to their physicochemical properties and route of administration. Indeed, anthelmintics are given orally and achieve high concentrations in the gastrointestinal tract but usually very low concentrations in the circulation [92]. Thus, one of the advantages of reprofiling—namely, knowledge of previous safety data—would not apply in case of systemic application. Furthermore, achieving sufficient efficacy of repurposed anthelmintics for cancer therapy may require treatment at higher doses and/or for longer periods as compared with the conventional indication, thereby resulting in unexpected side effects. In the effort to circumvent these issues, strategies aimed at increasing solubility and dissolution rate of anthelmintic drugs (e.g., nanocrystals and lipid-based formulations) [93] are being tested in order to improve their oral absorption and reach therapeutic blood levels.

## 4. Conclusion

Collectively, the available data indicate that the repositioning of anthelmintic agents in the treatment of tumors of the digestive system has promise and may represent a cheaper and faster route for expanding the arsenal of approved drugs. However, most, if not all of the above-mentioned hurdles should be addressed in order to have a realistic understanding of whether a repurposing project is a worthwhile opportunity and to increase the probability of success in clinical trials.

## Figures and Tables

**Table 1:** Anti-tumor effects of anthelmintic drugs in malignancies of the digestive apparatus.

Drug	Cancer Type	Observation	Ref.
Mebendazole	Gastric cancer	Inhibition of cell growth and invasion of a human malignant cell line derived from a primary gastric tumor, whether used alone or in combination 5-FU	[23]
Mebendazole	CRC	Cytotoxic activity against CRC cell lines (HCT-116, RKO, HT29, HT-8 and SW626)	[24]
Mebendazole	CRC	Reduction of the number and size of intestinal microadenomas in *Apc*^min/+^ mice, once used in combination with sulindac, through the inhibition of MYC and COX2 pathways, cytokine release and angiogenesis	[25]
Mebendazole	CRC	Induction of lung and lymph node metastases remission as well as partial liver metastases remission in a patient with refractory metastatic colon cancer	[26]
Mebendazole	HCC	Inhibition of the MAPK pathway in vitro and in vivo, whether used alone or in combination with Sorafenib, and improvement of liver function in an animal model of HCC	[27]
Albendazole	CRC	Cytostatic effect on HT29 cells in vitro and reduction of cancer growth in nude mice bearing intraperitoneal HT29-derived tumors	[28]
Albendazole	CRC	Anti-proliferative effects on SW480, SW620, HCT8 and Caco2 cells, especially when used in combination with paclitaxel	[29]
Albendazole	HCC	Cytostatic effects on rat, mice and human HCC cells. Reduction of tumor growth in nude mice inoculated with SK-HEP1 cells	[30]
Albendazole	Pancreatic cancer	Cytostatic and pro-apoptotic effects on PANC-1 and SW1990 cells in vitro and in a mouse xenograft model	[31]
Flubendazole	CRC	Anti-proliferative effect on SW480, SW620, HCT8, and Caco2 cell lines, also in combination with paclitaxel. Such effect was associated with cyclin B1 and cyclin D1 down-regulation	[29,32]
Flubendazole	CRC	Impairment of phosphorylation/activity of NF-kB in SW480 and SW620 cell lines, suppression of the expression of metastatic markers as well as cell migration	[33]
Niclosamide	CRC	Inhibition of the Wnt/β-catenin signaling pathway in the human CRC cell lines HCT-116, HT-29 and Caco2 by down-regulation of Dishevelled-2. Anti-cancer effects in CRC cells isolated by surgical resection of metastatic disease as well as in NOD/SCID mice implanted with human CRC cell-derived xenografts	[34]
Niclosamide	CRC	Anti-cancer effects on CRC mediated by the induction of autophagy	[35]
Niclosamide	CRC	Anti-proliferative and pro-apoptotic effects on SW480, SW620 and CC531 cells by affecting the formation of β-catenin-Bcl9-LEF/TCF triple-complex and inducing c-jun expression	[36]
Niclosamide	HCC	Cell growth inhibition and induction of apoptotic cell death in HepG2 and QGY7701 cell lines by eliciting ER stress	[37]
Niclosamide	HCC	Impairment of proliferation and induction of apoptosis in HepG2, QGY-7703 and SMMC-7721 cell lines by negatively affecting the phosphorylation/activity of the oncogenic transcription factor STAT3	[38]
Niclosamide	Esophageal cancer	Suppression of STAT3 signaling pathway resulting in the arrest of esophageal adenocarcinoma cells (BE3) and esophageal squamous cell carcinoma cells (CE48T and CE81T) in the G0/G1 phase of the cell cycle	[39]
NiclosamideEthanolamine	HCC	Cytostatic effect on HCC cells and impairment of cell migration, whether used alone or in combination with oxyclozanide	[40]
Niclosamide Ethanolamine	CRC	Reduction of intestinal polyp formation in *Apc*^min/+^ mice. Decrease of hepatic metastasis in a mouse model of CRC metastasis	[40]
Rafoxanide	CRC	Selective induction of ER stress in HCT-116 and DLD1 cells associated with cyclin D1 protein down-regulation, accumulation of cells in the G0/G1 phase and subsequent caspase-dependent apoptosis. Ani-mitogenic effect in human CRC explants. Reduction of both number and size of neoplastic lesions in *Apc*^min/+^ mice	[41]
Rafoxanide	CRC	Induction of autophagy and DAMPs (i.e., ecto-calreticulin exposure, ATP/HMGB1 release) in HCT-116 and DLD1 cells, resulting in immunogenic cell death. Reduction of tumor growth in vaccination experiments in vivo using immunocompetent mice and syngeneic cancer cells	[42]
Rafoxanide	Gastric cancer	Arrest of gastric cancer cells in the G0/G1 phase of the cell cycle, induction of autophagy and apoptosis through the inhibition of the PI3K/Akt pathway both in vitro and in vivo	[43]
Closantel	Liver cancer, pancreatic cancer	Cytostatic effect in zebrafishes xenotransplanted with human liver and pancreatic cancer cells	[44]
Nitazoxanide	CRC	Induction of apoptosis in CRC cell lines in a GSTP1-dependent manner	[45]
Nitazoxanide	CRC	Inhibition of mitochondrial respiration and mTOR pathway in HCT-116- and HT-29-derived spheroids. Suppression of tumor growth in combination with Irinotecan in a mouse xenograft model	[46]
Nitazoxanide	CRC	Anti-cancer activity on CRC cells via the impairment of Wnt/β-catenin signaling pathway	[47]
Nitazoxanide derivative(RM4819)	CRC	Cell cycle arrest and suppression of mitochondrial complex III activity. Inhibition of the proliferation of intestinal tumoroids	[48]
Ivermectin	CRC	Inhibition of Wnt-TCF pathway in DLD1 and Ls174T cells. Blockade of colon cancer stem cell self-renewal. Impairment of the growth of DLD1- and HT-29-derived xenografts in nude mice in a TCF-dependent fashion	[49]
Ivermectin	Gastric cancer	Suppression of MKN1 cell growth in vitro and in vivo through the inhibition of the nuclear expression of YAP1	[50]
Ivermectin	CCA	Induction of S-phase cell cycle arrest and apoptotic cell death in both gemcitabine-sensitive (KKU214) and gemcitabine-resistant (KKU214GemR) CCA cell lines	[51]
Praziquantel	CRC	Synergistic negative effect on DLD1 cell growth and viability, associated with XIAP down-regulation, in combination with paclitaxel	[52]
Pyrvinium pamoate	Pancreatic cancer	Cytotoxic effect on PANC-1 cells cultured under glucose starvation associated with the inhibition of Akt phosphorylation. Anti-tumor activity in vivo in a hypovascular pancreatic cancer model where immunocompromised mice were xenografted with PANC-1 cells	[53]
Pyrvinium pamoate	Pancreatic cancer, CRC	Impairment of glucose starvation-driven transcriptional activation of UPR-related genes (e.g., *GRP78*, *GRP94*, *XBP-1*, *ATF4*). Anti-tumor activity in nude mice transplanted with either HCT-116 or the pancreatic cancer cell line AsPC-1 in combination with doxorubicin	[54]
Pyrvinium pamoate	CRC	Inhibition of Wnt/β-catenin signaling in HCT-116 and SW480 cells via interaction with CK1α and pygopus down-regulation	[55]
Pyrvinium pamoate	CRC	Reduction of intestinal adenoma formation in *Apc*^min/+^ mice through the inhibition of the Wnt signaling	[56]
Pyrvinium pamoate	CRC	Synergistic anti-cancer effect on HCT-116 and SW620 cell lines in combination with 5-FU. Inhibition of the Wnt signaling in HCT-116 and SW620 cells as well as in human CRC explants. Impairment of liver metastases formation in nude mice injected with HCT-116 in the portal vein	[57]
Piperazine derivative(AK301)	CRC	Impairment of tubulin polymerization and induction of mitotic arrest in HT-29 and HCT116 cells. Increase of the susceptibility to TNF-α-mediated apoptosis	[58]
Piperazine derivative(BK1000S7)	HCC	Blockade of HepG2 and SK-Hep1 cell growth upon cyclin D1 down-regulation. Induction of apoptosis via caspase-3 and PARP-1 protein cleavage, impairment of AKT/ERK kinase phosphorylation and survivin expression	[59]

Abbreviations: CRC: colorectal cancer; 5-FU: 5-fluorouracil; APC: adenomatous polyposis coli; COX: cyclooxygenase; HCC: hepatocellular carcinoma; MAPK: mitogen-activated protein kinase; NF-kB: nuclear factor kB; NOD: non-obese diabetic; SCID: severe combined immunodeficient; LEF: lymphoid enhancer factor; TCF: transcription factor T-cell factor; ER: endoplasmic reticulum; STAT3: signal transducer and activator of transcription 3; DAMP: damage-associated molecular pattern; ATP: adenosine triphosphate; HMGB1: high mobility group box 1; PI3K: phosphatidylinositol 3-kinase; GSTP1: glutathione-S-transferase P1; mTOR: mammalian target of rapamycin; YAP1: yes-associated protein 1; CCA: cholangiocarcinoma; CK1: casein kinase 1; TNF: transforming necrosis factor; PARP: poly(ADP-ribose) polymerase; ERK: extracellular signal-regulated kinase.

**Table 2:** Anthelmintic drugs employed in clinical trials to treat cancers of the digestive system.

Drug	Cancer Type	Title of the Study	Phase	Identifier/Ref.
Mebendazole	CRC	Clinical study evaluating Mebendazole as adjuvant therapy in patients with colorectal cancer	2	NCT03925662
Mebendazole	Gastrointestinal cancer	Cytotoxic activity against five CRC cell lines	Terminated (lack of effect)	NCT03628079
Niclosamide	CRC	A Phase I study of Niclosamide in patients with resectable colon cancer	Terminated (low accrual)	NCT02687009
Niclosamide	CRC	Phase II Trial to investigate the safety and efficacy of orally applied Niclosamide in patients with metachronous or synchronous metastases of colorectal cancer progressing after therapy	2	NCT02519582
Niclosamide	FAP	The chemopreventive effect of Niclosamide in patients with familial adenomatous polyposis: double blinded randomized controlled study	2	NCT04296851
Levamisole	CRC	Levamisole and Fluorouracil for adjuvant therapy of resected colon carcinoma	Terminated	[60]
Levamisole	CRC	Prospectively randomized trial of postoperative adjuvant chemotherapy in patients with high-risk colon cancer	Terminated (lack of effect)	[61]
Levamisole	CRC	Clinical trial to assess the relative efficacy of fluorouracil and leucovorin, fluorouracil and levamisole, and fluorouracil, leucovorin and levamisole in patients with Dukes’ B and C carcinoma of the colon: results from National Surgical Adjuvant Breast and Bowel Project C-04	Terminated (lack of effect)	[62]
Levamisole	HCC	Multicenter, randomized, open, parallel, prospective, exploratory clinical study of Arginine Hydrochloride and levamisole in the treatment of advanced HCC	3	NCT03950518
Levamisole	Intrahepatic CCA	The efficacy of Levamisole Hcl in advanced intrahepatic cholangiocarcinoma. A multicenter, open, randomized, prospective study	3	NCT03940378

Abbreviations: CRC: colorectal cancer; FAP: familial adenomatous polyposis; HCC: hepatocellular carcinoma; CCA: cholangiocarcinoma.
